# Cytotoxicity  of dental cement on soft tissue associated with dental implants at different time intervals

**DOI:** 10.12688/f1000research.140071.1

**Published:** 2023-10-16

**Authors:** Prashanth Bajantri, Shobha J. Rodrigues, Shama Prasada Kabekkodu, Akshar Bajaj, Puneeth Hegde, Sandipan Mukherjee, Sharon Saldanha, Mahesh Mandatheje, Thilak Shetty B, Umesh Y. Pai, Ann Sales, Vignesh Kamath

**Affiliations:** 1 Department of Prosthodontics and Crown and Bridge, Manipal College of Dental Sciences, Mangalore, Manipal Academy of Higher Education, Manipal, Karnataka, 576104, India; 2 Cell and Molecular Biology, Manipal Academy of Higher Education Manipal, Manipal, Karnataka, 576104, India; 3 Department of Biomaterials and Restorative Sciences, Henry M Goldman School of Dental Medicine Boston University, United States Of America, United States Of America, USA

**Keywords:** Gingival fibroblast, implants, MTT assay, Luting cements, Resin

## Abstract

**Background:** To investigate and compare the effect of four commercially used dental cement at 24 hours, 48 hours,72 hours (h) and 6 days on the cellular response of human gingival fibroblast (HGF).

**Methods:** 3 cement pellet samples were made for each 4-test cement (n=12). The cement used for this study were zinc phosphate (ZP), zinc oxide non-eugenol (ZOE), RelyX U200 (RU200), and glass ionomer cement (GIC). The cytotoxicity of peri-implant tissues was investigated using one commercial cell line. All processing was done following International Organization for Standardization (ISO) methods 10993-5 and 10993-12 (MTT assay Test). Cell cultures without dental cement were considered as control. Standard laboratory procedures were followed to permit cell growth and confluence over 48 hrs after sub-cultivation. Before being subjected to analysis, the cells were kept in direct contact with the cement samples for the suggested time period. To validate the results the specimens were tested three times each. Cell death and inhibition of cell growth were measured quantitatively. Results were analyzed using 1-way ANOVA (a=0.05) followed by Tukey B post hoc test.

**Results:** The study showed the dental cement test material was cytotoxic. ZOE, ZP, GIC, and RU200 were cytotoxic in decreasing order, respectively, significantly reducing cell viability after exposure to HGF (p <0.001).

**Conclusions:** Within the limitations of this in-vitro cellular study, results indicated that HGF were vulnerable to the test the dental cement. The highest cytotoxicity was observed in ZOE, followed by ZP, GIC, and RU200.

## Introduction

Endosseous implants to replace lost teeth have become the gold standard in dentistry. The implant-supported prosthetic reconstruction uses both cement- and screw-retained restorations. Regarding cost, aesthetics, ease of fabrication and passivity, cement-retained implant supported prosthesis (CRISP) restorations function better than screw-retained ones. ^1^

Dental professionals often choose dental cement for CRISP based on preferences of the product’s characteristics, such as material properties, mixing method, delivery system, or consistency. ^2^^–^^4^ There are many kinds of luting dental cement that can be usedto lute bridges, crowns, veneers, and implant crowns, and dental materials that are biocompatible are gaining more attention from dentists and patients. ^3^^,^^4^ CRISP have both advantages and disadvantages. One of them is related to the leftover cement in subgingival areas. ^5^^–^^7^ In these instances, it is very hard to remove all the dental cement. ^8^ Peri-implant diseases are complex, inflammatory conditions caused by a group of bacteria that are usually anaerobic and Gram-negative and proliferate subgingivally. ^9^^–^^17^ One of the most important aspects of implant therapy success is the type of luting cement used to bond prostheses. ^18^ It has been proved that residual cement left in CRISP can be an etiological factor for peri-implantitis in 8.6-14.4% of cases. ^19^^–^^23^

ZP cement is a dental cement that has been shown to be very effective in dental practice. ^24^^–^^27^ However, it has some disadvantages, such as being soluble, low pH, and inability to bond with the tooth chemically. Zinc oxide non eugenol (ZOE) is a temporary cement that uses different substitutes instead of eugenol, because eugenol can interfere with resin bonding. Traditional GICs have some benefits, such as being biocompatible, releasing fluoride, having a thermal expansion and an elasticity similar to dentin. ^28^

Traditional GICs have drawbacks such as dehydration, susceptibility, high solubility, and slow setting rate despite their benefits. Due to further developments in GICs, resin-modified GICs—which have greater physical and mechanical properties than regular GICs—have been introduced. ^29^ Resin cement includes a significant amount of composite resin, which chemically attaches to the tooth. ^30^ Despite their biocompatibility issues, dentists have been increasingly using this cement since they are said to improve restorative retention. However, certain material components can triggers a pulpal or gingival reaction, raising concerns about cytotoxicity. ^29^^,^^31^^–^^33^ Furthermore, despite GICs' increased mechanical qualities, only a few studies have shown their biocompatibility and harmful consequences. ^29^^,^^30^

Dental cement can significantly influence the growth or suppression of various bacterial strains associated with peri-implant disease. Furthermore, the effect of dental cements on the proliferation of host cells can provide additional insights for choosing the appropriate cement material.

In clinical scenarios where cement might be applied during the early healing phase, such as temporary restoration or abutment placement during surgery, or at a later stage in implant restoration where harmful bone alterations might occur if cement comes into contact with host tissues, it's particularly crucial to assess the effects of bacteria and host cells on dental component surfaces. ^34^

Bone loss around dental implants typically follows a specific pattern, moving from the crestal bone level towards the apex of the implant. This resorption profile may be primarily due to the interaction between soft tissue and remnants of cement from cement-retained prosthesis. ^35^

Raval et al. conducted a study on the bacterial response of many late-stage Gram-negative colonisers, which are implicated in peri-implant disease. ^36^

Different cement formulations (zinc oxide with eugenol, zinc oxide with noneugenol, zinc phosphate, and resin components), according to the authors' hypotheses, would produce various bacterial reactions. Several commercially available dental cements were exposed to Aggregatibacter actinomycetemcomitans, Fusobacterium nucleatum, and Porphyromonas gingivalis, and the bacterial viability was monitored to ascertain how the bacteria responded to the dental cement. The composition of dental cement has a substantial influence on whether certain bacterial strains linked to peri-implant illness proliferate or are inhibited. A previous study by the authors studied the impact of dental cement on cell proliferation at 24 hrs. ^37^ However, the impact of dental cement on host cellular proliferation in different time intervals may throw some light in choosing the right cement material as these cement may remain in contact with host tissues for varied time periods depending on the purpose and duration of the restoration.

Several factors may restrict the application of in vitro cell-based experiments to clinical scenarios. Primary cells, which maintain the same natural ploidy, gene expression regulation, stress response, and other biological parameters seen in humans, may provide more relevant data for clinicians compared to immortalised cell lines. ^38^

Human gingival fibroblasts are commensals of the oral environment and make interesting models for invitro evaluation of dental materials on account of the release of growth factors and cytokines suggesting immunological response. The current study therefore employed human gingival fibroblasts.

This study investigated and compared the effect of four commercially used dental cement at 24h, 48h, 72h, and six days on human gingival fibroblast (HGF) cellular response. The Null Hypothesis was that commercially used dental cement would have had no effect on the cellular response of HGF.

## Methods

The test cement used in the study is presented in Table 1.

**Table 1.** Test cement used in the study.

Cement type (Groups)	Product name	Manufacturer
Zinc phosphate	De Trey Zinc	Dentsply Sirona
Zinc oxide non Eugenol	Rely X ^TM^ Temp NE	3M ESPE
Resin cement	Rely X ^TM^ U 200	3M ESPE
Glass ionomer	GC Gold Label	GC I

### Preparation of the test cement

The preparation of the cement samples was done as follows: The powder was stirred into the liquid in modest increments as per the manufacturer's directions. Once the cement acquired the right consistency, it was transferred to three polytetrafluoroethylenes (PTFE) polymer moulds. The cement takes roughly 5-9 minutes to set.

### Glass Ionomer Cement (GIC)

The GIC powder and liquid were combined as per the manufacturer's instructions. The mixture was immediately transferred to three PTFE moulds to preserve the gel structure. The GIC cement was left to cure for 24 hours.

### Zinc oxide non-eugenol cement

The base and catalyst were mixed according to the manufacturer's guidelines on a mixing pad, then transferred to three PTFE moulds. The setting time for this cement was approximately 3 minutes and 30 seconds.

### Resin cement

The required amount of resin was dispensed from the automix syringe's clicker dispenser, mixed, and poured into three PTFE moulds. The RU200 cement set in about 30 seconds.

All tests used a mould of size 7x3x3mm. The cement was kept at room temperature for two days, then washed with phosphate buffer saline pH7.4, and air-dried in a NUAIR biosafety cabinet hood to clean the surface of the solidified cement and remove any unsolidified items.

### Cytotoxicity evaluation

The cytotoxicity of the four test cements on human gingival fibroblast (HGF) cells was assessed using the MTT assay Test [(3-(4, 5-dimethylthiazol-2-yl)-2, 5-diphenyltetrazoliumbromid], as previously described. The HGF cells were cultured in DMEM supplemented with 10% FBS and maintained in a class II biosafety cell culture hood (Nuair, USA) in a CO2 incubator (ThermoFisher Scientific, USA).

In brief, 1X104 HGF cells were grown in a 96-well plate and immediately exposed to the four test cements for a day at 37°C with 5% CO2. Direct contact testing was used to closely simulate an in vivo setting and assess the cytotoxicity of the dental cements under investigation. HGF cells unexposed to cement served as a control group. After the incubation period (24h, 48h, 72h, and six days), the cement was removed, and the cells were treated with MTT (5 mg/ml, Sigma, USA) for 4 hours at 37°C with 5% CO2. The formazan crystals formed were dissolved in 150 μL of DMSO (Sigma, USA), and the absorbance was measured using a microplate reader (Varioskan, ThermoFisher Scientific, USA) at 570 and 630 nm. All tests were performed in duplicate and repeated three times for validation.

### Statistical analysis

The experiments were conducted with appropriate replicates and repeated three times. Intergroup comparison was done using the post-hoc Tukey Test. Statistical significance was defined as P > 0.05 using the Tukey post-hoc test.

## Results

**Figure 1.**
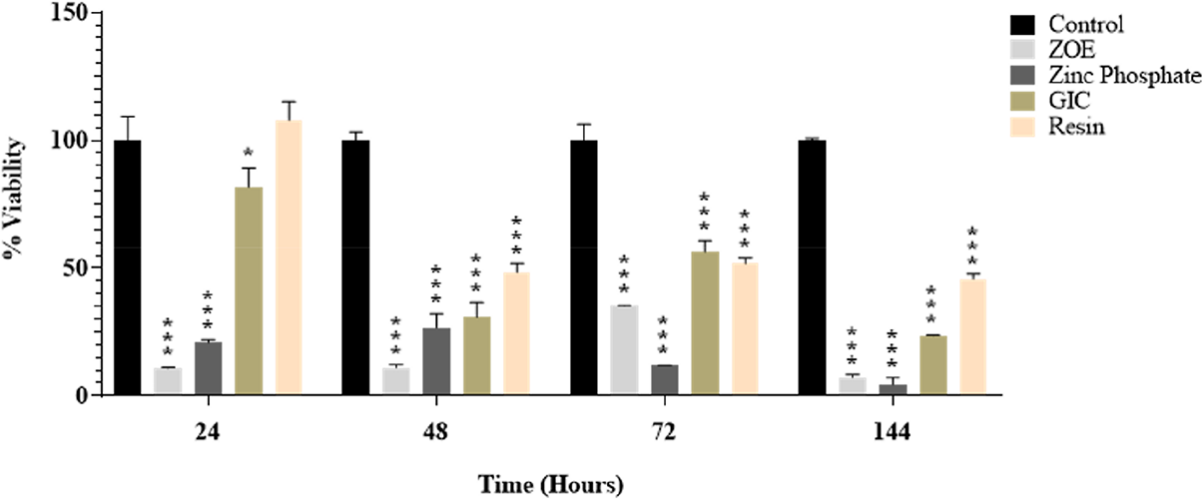
The effect of direct cement exposure on the viability of HGF cells at different time intervals.

The present study was undertaken to determine the cytotoxic effect of four different dental cement on HGF. Cell viability was measured 24hrs, 48hrs, 72hrs, and 6 days post-exposure with four dental cements. The intergroup comparison was carried out using post-hoc Tukey Test. *P* > 0.05 by Tukey post-hoc test was considered statistically significant. ( Figure 1)

### Cell viability at 24h time interval

HGF viability was 99.99% in the control cement at 24 hours, 10.84% in the ZOE cement, 20.82% in the ZP cement, 81.73% in the GIC, and 107.78% in the RU200 cement, respectively ( Figure 1). Compared to the other three cement it was tested, RU200 had the highest cell viability, and ZOE cement had the lowest cell viability. There is a statistically significant difference between all of the groups ( *P* <0.0001) except for the control and RU200 cement group ( *P*=0.5635).

### Cell viability at 48-hour time interval

HGF viability in control, ZOE, ZP, - GIC and RU200 was 99.99 percent, 11.03 percent, 26.76 percent, 30.79 percent, and 48.29 percent, respectively, after 48 hours. Compared to the other three cements, RU200 has the highest cell viability. Cell viability is lowest in ZOE and ZP cement. There is a statistically significant difference between all groups, P< 0.0012, except for the zinc phosphate and GIC group (P=0.7575).

### Cell viability at 72 hours time interval

HGF viability was 99.99 percent, 35.11 percent, 11.86 percent, 56.34 percent, and 51.71 percent at 72 hours in control, ZOE, ZP, GIC, and RU200, respectively. GIC has the most excellent cell viability compared to the other three cement groups, while ZP cement has the lowest. Of the four cement groups, ZOE and ZP cement had the lowest cell vitality. The results were statistically significant between all of the groups. **(P< 0.0001) except ZOE and ZP (p=0.7954) and ZOE and GIC(P=0.0669)**

### Cell viability at sixth day time interval

On 6 ^th^ day the HGF viability in control, ZOE, ZP, GIC cement and RU200 cement was 99.99%,7.14%,4.53%,23.51%, and 45.85%, respectively. RU200 cement shows the highest cell viability, while zinc oxide non-eugenol cement shows the least cell viability among all 4 cement. The results were statistically significant between all of the groups. **(P<0.0001) except for ZOE and ZP (P =0.7811)**

This study evaluated the cytotoxic impact of four distinct dental cement on Human Gingival Fibroblasts (HGF). The viability of the cells was assessed at 24 hours, 48 hours, 72 hours, and 6 days after exposure to the cements. The Tukey post-hoc test was used for intergroup comparisons. A p-value greater than 0.05 was deemed statistically significant ( Figure 1).

At the 24-hour mark, the viability of HGF was highest in the RU200 cement and lowest in the ZOE cement, compared to the other three cements tested. The difference between all groups was statistically significant (P <0.0001), except for the control and RU200 cement group (P=0.5635).

After 48 hours, RU200 cement continued to show the highest cell viability among the four cements. The lowest cell viability was observed in ZOE and ZP cement. All groups showed a statistically significant difference (P< 0.0012), except for the zinc phosphate and GIC group (P=0.7575).

At the 72-hour interval, GIC showed the highest cell viability among the four cements, while ZP cement showed the lowest. The results were statistically significant between all groups (P< 0.0001), except between ZOE and ZP (p=0.7954) and ZOE and GIC (P=0.0669).

On the sixth day, RU200 cement demonstrated the highest cell viability, while zinc oxide non-eugenol cement showed the least among all four cements. The results were statistically significant between all groups (P<0.0001), except for ZOE and ZP (P =0.7811).

## Discussion

The experimental arrangement of this research displayed that human gingival fibroblast displayed susceptibility to alterations in viability when subjected to commercial dental cement. Furthermore, these fibroblasts can trigger biological responses. The Null Hypothesis was invalidated due to significant fluctuations in fibroblast viability, which suggests that the proximity of cement to periimplant tissue during cementation plays a role in causing potential toxic tissue damage. The extent of this damage is proportional to the quantity of cement in proximity with oral tissues and the leaching of components. Variations in individual sensitivity could exist, emphasizing the crucial necessity of removal of excess cement. ^39^

The evaluation of the cytotoxic potential of dental cement was carried out using a standardized technique in this experimental investigation. Utilizing in-vitro cytotoxic assays offers straightforward control over experimental parameters that are complex to manage in in-vivo studies. ^40^^–^^42^

The impact of ZP, ZOE, GIC, and RU200 cement on HGF cells was investigated using the MTT test. Chemical constituents released from tooth restorative materials target fibroblasts. HGF cells were chosen for this study due to their ease of production and cultivation. These cells offer benefits in terms of in-vitro growth efficiency and result reproducibility. They yield findings comparable to primary human gingival fibroblasts, potentially serving as a model for in-vitro studies on gingival toxicity. ^43^^–^^45^

The MTT test, a widely recognized method for assessing cell vitality, was employed. In the current investigation, when the MTT assay was conducted on cell cultures exposed to the test cement for 24 hours, the highest cell viability, at 107.78%, was observed with resin cement at 24 hours 48 hours, and 66 days. Conversely, the lowest viability was found with ZOE and ZP cement, respectively. Significant differences were found between the control, ZOE, and ZP groups.

At all the various time intervals, ZOE and ZP cement exhibited the most pronounced cytotoxicity. ZOE cement samples displayed severe cell cytotoxicity, with cell viability measuring below 30%. In the case of ZP cement, cell viability stood at 10.84%, 11.03%, 11.86%, and 4.53% at 24, 48, 72 hours, and the sixth day, respectively. This occurrence can be attributed to the release of leachable substances from the materials, an impact that progressively diminishes and eventually falls below detectable levels after a span of six weeks. ^46^ A preceding investigation conducted by the researchers revealed GIC to exhibit the highest cytotoxicity. ^37^However, it's noteworthy that the time frame analyzed in that study was limited to 24 hours. ^37^ Discrepancies in methodology could play a role in yielding differing outcomes. Potential explanations for the cytotoxic impacts of these cements include the liberation of zinc and fluoride ions, as well as acidity and the discharge of other substances. ^27^

Leirskar and Helgeland conducted an analysis of the culture conditions involving ZP cement and observed the released zinc had detrimental effects on the studied cell line. In addition since cell death was noted from the first to the third day, it indicated that factors apart from acidification were incriminated. These findings aligned with research by Welker and Neupert, as well as Leirskar et al. ^40^^,^^41^

During cell death, proinflammatory cytokines generally increase, making the suppression of inflammatory responses a key aspect of effective anti-inflammatory ingredients. The extent of cytotoxic effects was linked to the quantity of zinc released from ZP cement, suggesting that other elements, like the acid produced by the cement, might amplify the zinc's impact. Earlier studies revealed that zinc absorption in certain cell types reduced as pH dropped. ^47^

Zn2+ acts as an anti-inflammatory ingredient by regulating proinflammatory cytokines, which is why a higher zinc dose is recommended to lower these cytokine levels in the blood plasma of inflammation patients. Another study found that Harvard ZP cement exhibited greater cytotoxicity compared to Fuji PLUS cement (RM-GIC). Fuji PLUS had varying levels of cytotoxic effects, ranging from mild to severe, while Fuji I GIC showed the least cytotoxicity.

Lewis et al. suggested that the leachable components of GICs might affect the rate of cell cycle progression rather than causing immediate cell death. ^47^According to Oliva et al., RM-GIC has notable cytotoxic effects attributed to its leaching poly-acidic phase. The higher HEMA content in Fuji PLUS cement which quickly diffuses into dentine might contribute to its greater cytotoxicity compared to Fuji I. ^22^^–^^24^

The levels of HEMA permeating into pulpal tissue are considerably lower than those causing acute toxicity. Thus, RM-GIC's cytotoxicity could be linked to the leaching of a hazardous mix of components, including resin monomers and fluoride ions. Kanjevac et al. established a connection between cytotoxicity and fluoride leakage in current GIC cement, also noting that other components like strontium and aluminum ions had more harmful effects on cell cultures when leached. Due to higher fluoride release, Fuji PLUS (RMGIC) exhibited more cytotoxicity than Fuji I (GIC). ^33^^,^^48^^,^^49^

The quantities of HEMA capable of permeating into pulpal tissue are notably lower than those causing acute toxicity. Consequently, the leaching of a hazardous mixture of components, including resin monomers and fluoride ions, could be suspected in the case of RM-GIC. ^21^^,^^22^ Kanjevac et al. conducted a study linking cytotoxicity to fluoride leakage in current GIC cement. ^44^ They also highlighted that other elements like strontium (Sr2+) and aluminum ions (Al3+) had more pronounced harmful effects on cell cultures when leached. Due to increased fluoride release, Fuji PLUS (RMGIC) exhibited greater cytotoxicity than Fuji I (GIC). ^50^^,^^51^

Previous in-vitro investigations have shown that when diffusates are extracted from cells using a dentin barrier test device, the apparent cytotoxicity of materials is reduced. ^52^ Dentin can absorb chemicals in the tubules and hinder the diffusion of harmful compounds into the pulp, as explained by Hanks et al. ^53^

Self-adhesive resin cements, composed of filled polymers, are designed to bond to tooth structure without requiring additional adhesives or etchants. These cements might undergo a slower rate of polymerization and attain a lower final polymerization degree compared to traditional resin cements, whether in dual- or self-cured modes, as noted by Moraes et al. ^54^ Their final polymerization degree often proved higher in the dual-cure mode. Dual-cured specimens of resin-based cement (Duo-Link and BisCem) were demonstrated to be more harmful than chemically set cement. BisCem exhibited more cytotoxicity than Rely-XTM Unicem in the study by Ulker and Sengun. ^55^ This aligns with Schmid-Schwap et al., who found that self-adhesive cement (Rely-XTM Plus) was more cytotoxic than adhesive resin cement. ^46^

However, certain monomers in resin composite cement can enter dentin tubules, potentially causing pulpal damage and hindering pulp tissue healing. ^56^^–^^58^ These monomers exhibit cytotoxicity in vitro for pulp and gingival cells, and ions could trigger cell changes. ^35^ Bakopoulou et al. demonstrated that the cytogenetic effects were caused by released chemicals like TEGDMA found in the composition of resin cement. The cytotoxicity ranking of commonly used monomers was identified as Bis-GMA > UDMA > TEGDMA > HEMA > MMA. ^57^^,^^59^^–^^61^ The cytotoxic effects of Duo-Link cement could be attributed to the presence of UDMA and inorganic fluoride. The most severe cytotoxic effects in this study were associated with BisCem composite resin cement, which comprises TEGDMA and HEMA. ^62^^,^^63^ Additionally, BisCem lowered the pH to 3-4, potentially explaining the decreased cell viability compared to Duo-Link cement's impact. Reduced drying time increases the cytotoxicity of resin cement, emphasizing the need for adequate curing time. ^64^

The findings also suggest that the choice of cell system may impact results in advanced biocompatibility assays, such as protein expression or -omics studies, or assessments of cell-cell interactions. This should be explored in future research with larger sample sizes for each experimental group. This is particularly relevant considering the need to prioritize more relevant and mechanistic insights into hazardous pathways over isolated cytotoxicity endpoints. Therefore, while the clinical relevance of in vitro assays needs careful consideration, the results of this study must be approached cautiously. Despite resin cement's strong retention and preferred status as a luting agent in CRISP, clinical reports indicate a high incidence of peri-implantitis and difficulties in prosthesis retrieval in complication cases. Thorough clinical practices are necessary to reduce soft tissue exposure to these cements during attempts to remove excess cement.

## Conclusion

Within the limitations of this in-vitro study, the following conclusions were drawn:

• All the luting cement is cytotoxic to the gingival fibroblast cells.
• Maximum cytotoxicity was demonstrated in ZOE, followed by ZP, GIC and RU200 cement.

## Data Availability

Figshare: Cytotoxicity of dental cements associated with dental implants, https://doi.org/10.6084/m9.figshare.23702505.v1. ^65^ Data are available under the terms of the Creative Commons Attribution 4.0 International license (CC-BY 4.0).
