# Supplementary material for: Cytotoxicity  of dental cement on soft tissue associated with dental implants at different time intervals
Source: F1000Res. 2023 Dec 12;12:1342. Originally published 2023 Oct 16. [Version 2] doi: 10.12688/f1000research.140071.2 (PMC11140311; doi:10.12688/f1000research.140071.2)
Supplement: Peer review history

# Reviewer report: Reviewer response for version 2

**DOI:** 10.5256/f1000research.159945.r284513

**Published:** 2024-06-03

**Authors:** Kannan Thirumulu Ponnuraj

**Affiliations:** 1 School of Dental Sciences, Universiti Sains Malaysia, Minden Heights, Pulau Pinang, Malaysia

The article has scientific merit. However, the corrections as indicated and questions posed in the manuscript using annotations need to be addressed. The repetition of information in the results section should be deleted. The source of HGF should be mentioned. The results section should be written in past tense. Note other minor corrections in grammar.

 Please view the additional review comments in the file found here.

Is the work clearly and accurately presented and does it cite the current literature?

Yes

If applicable, is the statistical analysis and its interpretation appropriate?

Yes

Are all the source data underlying the results available to ensure full reproducibility?

Yes

Is the study design appropriate and is the work technically sound?

Yes

Are the conclusions drawn adequately supported by the results?

Yes

Are sufficient details of methods and analysis provided to allow replication by others?

Yes

Reviewer Expertise:

Genotoxicity, Cytotoxicity, Mutagenicity

I confirm that I have read this submission and believe that I have an appropriate level of expertise to confirm that it is of an acceptable scientific standard, however I have significant reservations, as outlined above.

# Reviewer report: Reviewer response for version 2

**DOI:** 10.5256/f1000research.159945.r234384

**Published:** 2024-05-30

**Authors:** Bheema setty Manasali

**Affiliations:** 1 Taif University, Taif, Saudi Arabia

“Cytotoxicity of dental cement on soft tissue associated with dental implants at different time intervals”

 In my opinion, there isn’t much literature on the topic and the article should be well-received. Moreover, it studies the impact of dental cement on soft tissue in association with implants which is most relevant clinically.

 I also notice vast improvements in the writing style, recent references, and discussion.

 I notice that the discussion has been augmented with additional references, particularly contradictory findings.

 I do not have any major corrections to be suggested.

 However, as minor recommendations, I would be happy to see the authors suggesting clinical tips when dealing with resin cement considering the incidence of peri-implantitis.

 I recommend this article for approval.

 Thanking You

Is the work clearly and accurately presented and does it cite the current literature?

Yes

If applicable, is the statistical analysis and its interpretation appropriate?

Yes

Are all the source data underlying the results available to ensure full reproducibility?

Yes

Is the study design appropriate and is the work technically sound?

Yes

Are the conclusions drawn adequately supported by the results?

Yes

Are sufficient details of methods and analysis provided to allow replication by others?

Yes

Reviewer Expertise:

implants

I confirm that I have read this submission and believe that I have an appropriate level of expertise to confirm that it is of an acceptable scientific standard.

# Reviewer report: Reviewer response for version 1

**DOI:** 10.5256/f1000research.153400.r218095

**Published:** 2023-11-20

**Authors:** Roseline Meshramkar

**Affiliations:** 1 Shri Dharmasthala Manjunatheshwara College of Dental Sciences, Dharwad, India

The study is a meticulously crafted introduction, thorough exploration of materials and methods, and a comprehensive presentation of results. However, it is suggested that the discussion section be augmented with additional references, particularly those that present contradictory findings.

 Clinical applications of cements are ubiquitous, and their biocompatibility is of paramount importance. Despite the widespread clinical use of cements, the findings of this study warrant careful consideration. Notably, the results strongly advocate against the use of any cement for prosthetic applications. Consequently, it is imperative to address the clinical acceptability of utilizing cements in such contexts. To bolster the discussion and it is recommended to incorporate references that elucidate contrasting perspectives and outcomes.

 In conclusion, the study underscores the need for a cautious approach to the use of cements in prosthesis, challenging conventional clinical practices.

**Adding references** in the discussion makes the study more thorough and helps us understand the bigger picture of cytotoxicity of cements and its clinical relevance.

Is the work clearly and accurately presented and does it cite the current literature?

Yes

If applicable, is the statistical analysis and its interpretation appropriate?

Yes

Are all the source data underlying the results available to ensure full reproducibility?

Yes

Is the study design appropriate and is the work technically sound?

Yes

Are the conclusions drawn adequately supported by the results?

Yes

Are sufficient details of methods and analysis provided to allow replication by others?

Yes

Reviewer Expertise:

Esthetics, Smile design, Implants.

I confirm that I have read this submission and believe that I have an appropriate level of expertise to confirm that it is of an acceptable scientific standard.

## References

[1] : An Evaluation of the Effectiveness of Various Luting Cements on the Retention of Implant-Supported Metal Crowns. *Cureus* .2023;15(7) : 10.7759/cureus.41691 e41691 10.7759/cureus.41691 37575823 PMC10413795

# Response

**Published:** 2023-11-21

**Authors:** Dr Prashanth Bajantri

**Affiliations:** Prosthodontics crown and bridg, Manipal University, Mangalore, Karnataka, India

User Comment,

 Thank you very much Madam for your critical appraisal of our article .

 As suggested by you indeed Cements are ubiquitously used in prosthodontics and as per our study, they need to be cautiously considered in implant-related prosthesis.

 This as you suggest warrants additional references and we are happy to work on this.

 we thank you again for the time and effort you have invested in providing your  constructive critical  appraisal

 Our kind regards,

 Dr Prashant

# Response

**Published:** 2023-12-08

**Authors:** Dr Prashanth Bajantri

**Affiliations:** Prosthodontics crown and bridg, Manipal University, Mangalore, Karnataka, India

As suggested by the first reviewer we have made modifications to the manuscript to increase the number of references and to augment the discussion. Please find the edited manuscript. We look forward to the early indexing of this article on account of our professional compulsions. Kindly look into this matter.

 Kind regards,

 Shobha Rodrigues
